# Intraepineurial fat quantification and cross-sectional area analysis of the sciatic nerve using MRI in Charcot-Marie-Tooth disease type 1A patients

**DOI:** 10.1038/s41598-021-00819-0

**Published:** 2021-11-02

**Authors:** Hyun Su Kim, Ji Hyun Lee, Young Cheol Yoon, Min Jae Cha, Soo Hyun Nam, Hye Mi Kwon, Seonwoo Kim, Hojeong Won, Byung-Ok Choi

**Affiliations:** 1 Department of Radiology, Samsung Medical Center, Sungkyunkwan University School of Medicine, Seoul, South Korea; 2 Department of Radiology, Chung-Ang University Hospital, Chung-Ang University College of Medicine, Seoul, South Korea; 3 Department of Neurology, Samsung Medical Center, Sungkyunkwan University School of Medicine, 81 Irwon-ro, Gangnam-gu, Seoul, 06351 South Korea; 4 Statistics and Data Center, Research Institute for Future Medicine, Samsung Medical Center, Sungkyunkwan University School of Medicine, Seoul, South Korea; 5 Department of Health Sciences and Technology, SAIHST, Sungkyunkwan University, Seoul, South Korea

**Keywords:** Neurology, Demyelinating diseases, Musculoskeletal system

## Abstract

The objectives of this study were to assess the fat fraction (FF) and cross-sectional area (CSA) of the sciatic nerve in Charcot-Marie-Tooth disease type 1A (CMT1A) patients using Dixon-based proton density fat quantification MRI and to elucidate its potential association with clinical parameters. Thigh MRIs of 18 CMT1A patients and 18 age- and sex-matched volunteers enrolled for a previous study were reviewed. Analyses for FF and CSA of the sciatic nerve were performed at three levels (proximal to distal). CSA and FF were compared between the two groups and among the different levels within each group. The relationship between the MRI parameters and clinical data were assessed in the CMT1A patients. The CMT1A patients showed significantly higher FF at level 3 (*p* = 0.0217) and significantly larger CSA at all three levels compared with the control participants (*p* < 0.0001). Comparisons among levels showed significantly higher FF for levels 2 and 3 than for level 1 and significantly larger CSA for level 2 compared with level 1 in CMT1A patients. CSA at level 3 correlated positively with the CMT neuropathy score version 2 (CMTNSv2). In conclusion, the sciatic nerve FF of CMT1A patients was significantly higher on level 3 compared with both the controls and the measurements taken on more proximal levels, suggesting the possibility of increased intraepineurial fat within the sciatic nerves of CMT1A patients, with a possible distal tendency. Sciatic nerve CSA at level 3 correlated significantly and positively with CMTNSv2, suggesting its potential value as an imaging marker for clinical severity.

## Introduction

Charcot-Marie-Tooth disease (CMT) is a group of clinically and genetically heterogeneous inherited neuromuscular disorders that are characterized by symmetric distal muscle wasting, weakness, and sensory loss^1,2^. The most common form, CMT type 1A (CMT1A), which constitutes about 40% of all CMT cases, results from duplication of the peripheral myelin protein 22 (PMP22) gene on chromosome 17^3^. CMT1A is a demyelinating neuropathy, that typically demonstrates hypertrophy of nerve fascicles through a marked increase in endoneurial collagen and frequent onion-bulb formations^4–6^. Nerve hypertrophy in CMT1A patients has been demonstrated as an increased cross-sectional area (CSA) on imaging studies compared with normal controls^4,7–9^.

The myelin sheath contains a high proportion (70–85%) of lipids, and its formation and maintenance require a high level of lipid synthesis and extracellular uptake^10^. Studies using a rat model of CMT1A have reported that Schwann cells in this disease exhibit reduced transcription of the genes required for myelin lipid biosynthesis^11–14^. This perturbed lipid metabolism could be involved in the pathogenesis of the disease by reducing lipid incorporation into myelin, which produces structural changes in the myelin sheath^11^. Moreover, a lipid-enriched diet has been shown to improve myelination in CMT animal models^11,13^, which suggests that exogenous lipid delivery to the peripheral nerves via plasma could potentially affect the myelination of nerve fibers in this disease.

Interfascicular fat constitutes the major portion of fat within the epineurium of the peripheral nerves^15^. But it has not been thoroughly investigated with regard to the abnormal lipid metabolism of myelin in CMT1A patients. The status of interfascicular fat in CMT1A patients, whether it is increased or decreased compared with normal subjects, has not been established. A few studies using magnetic resonance imaging (MRI) have suggested that an increase in interfascicular fat content within peripheral nerves could occur in CMT patients^16,17^. Nonetheless, although intraepineurial lipids in CMT1A patients are gaining increasing attention, the question remains underexplored.

Recently, MRI with various up-to-date imaging techniques has been applied to neuromuscular evaluation and attempts to derive potential imaging biomarkers for CMT1A patients^17–20^. Among them, Dixon MRI is an imaging technique increasingly used for fat fraction (FF) measurement. It has proved to be highly sensitive and reproducible for evaluating disease progression in the skeletal muscles of neuromuscular disorder patients^21–23^. Recent Dixon-based proton density fat quantification techniques provide FF maps that enable direct quantitative FF measurement within a designated region of interest (ROI)^24^. The study of Ratner et al. opened the possibility of applying this imaging technique to peripheral nerve structures^25^. They found Dixon-based MRI to be a reliable tool for measuring FF, mostly representing interfascicular fat, in the sciatic nerves of normal subjects. However, measuring intraepineurial FF using Dixon-based MRI in neuromuscular disorders such as CMT has not been reported to our knowledge.

Our purpose in this study was to evaluate the fat within the sciatic nerves of CMT1A patients by comparing the sciatic nerve FF, acquired using Dixon-based proton density fat quantification MRI data, of CMT1A patients with that of healthy controls. We also measured and compared the sciatic nerve CSA between the two groups and examined whether those MRI parameters were correlated with clinical parameters.

## Methods

### Study subjects

Our institutional review board (Samsung Medical Center, IRB File No. 2020-07-108) approved this study and waived the requirement for informed consent. The study was conducted in accordance with the declaration of Helsinki. MRI and clinical data from subjects prospectively enrolled between February and June 2017 for a previous study (18 CMT1A patients and 18 age- and sex-matched volunteers) were retrospectively used for this analysis^21^. All the subjects gave written informed consent to the previous study. Among the 18 CMT1A patients, 17 received a genetic analysis prior to MRI acquisition, and one patient received the analysis after MRI acquisition. All 18 patients were confirmed to have PMP22 duplication. The healthy volunteers were examined by a neurologist prior to the MRI to ensure the absence of any neurological abnormality.

### MRI acquisition

MRI was obtained using a 3.0 T MRI system (Ingenia; Philips Healthcare, Best, the Netherlands) with a 16-channel anterior coil and posterior built-in coil. The following imaging sequences were obtained for morphologic imaging: axial and coronal T1-weighted turbo spin echo sequences and the axial T2-weighted Dixon sequence. From the Dixon sequence, water-only, fat-only, in-phase, and out-of-phase images were generated. The MRI protocols are detailed in Supplementary Table S1.

A 3D multiple gradient echo Dixon-based proton density fat quantification sequence (mDixon-Quant; Philips Healthcare, Best, the Netherlands) was used to acquire the FF measurement, which sampled six-echo data. Images were obtained in the axial plane for the pelvic girdle and the thighs at levels from the anterior inferior iliac spine through the femur distal end. Water- and fat-only images were sequentially reconstructed, which automatically generated FF maps.

### Image analysis

**Figure 1:**
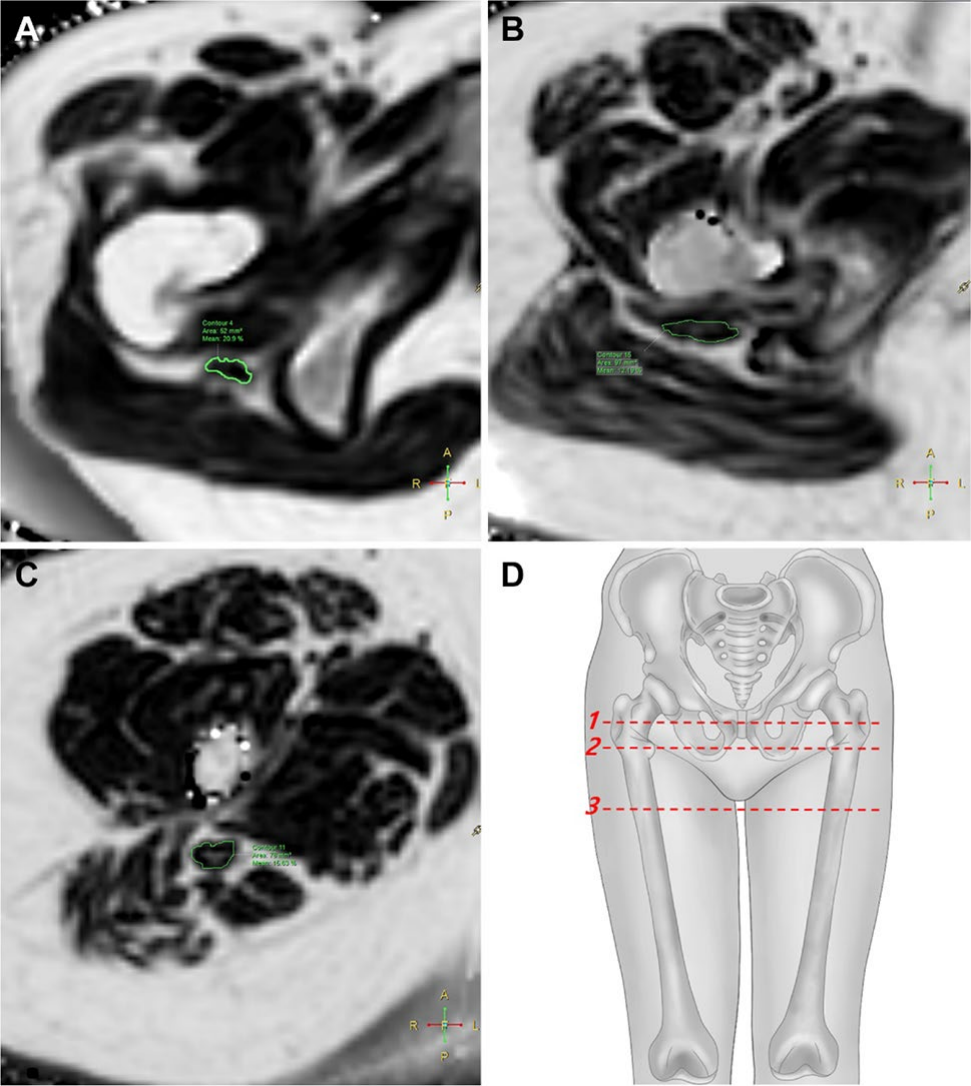
An example region of interest (ROI) analysis of the sciatic nerve in a 20-year-old female Charcot-Marie-Tooth disease type IA patient. Axial fat fraction maps of the 3D multiple gradient echo Dixon-based MRI (**A–C**) where the ROI analyses of the sciatic nerve were performed: hamstring tendon origin ((**A**), level 1), lesser trochanter of the femur ((**B**), level 2), and gluteus maximus tendon insertion ((**C**), level 3). The ROIs are indicated on the fat fraction maps showing the measured cross-sectional area and fat fraction values for the sciatic nerve. An illustration (**D**) shows the three levels where image analyses were performed.

Two radiologists (H.S.K. and J.H.L., with 4 years and 6 years of experience in musculoskeletal radiology, respectively) who were blinded to the clinical information independently performed image analysis using image-processing software (IntelliSpace Portal, version 10.1; Philips Healthcare). Each radiologist drew an ROI along the boundary of the sciatic nerve on the FF maps of the 3D multiple gradient echo Dixon-based MRI. The ROIs were carefully drawn to fall within the boundary of the nerve, avoiding the inclusion of extra-neural tissue, using the axial T1- and T2-weighted images and the water-only image from the 3D multiple gradient echo Dixon-based MRI as references. Analyses were performed on each side of the sciatic nerve at three levels (Fig. 1): hamstring tendon origin (level 1), where the uppermost part of the semimembranosus tendon is visualized; the lesser trochanter of the femur (level 2), where it is visualized most prominently; and gluteus maximus tendon insertion (level 3), where the uppermost part of the tendon insertion is visualized. We acquired the CSA and FF of each ROI using these analyses.

### Clinical assessments

Clinical data for the 18 CMT1A patients, onset age, CMT neuropathy score version 2 (CMTNSv2), 10 m (m) walk test, and 9-hole peg test, were used in the correlational analysis with the imaging parameters. Onset age was determined by asking the patient when symptoms such as distal muscle weakness, foot deformity, and/or sensory changes initially appeared. CMTNSv2 is a composite scoring system based on neurological symptoms, clinical signs, and electrophysiological parameters. It ranges from 0 (no deficit) to 36 (maximal deficit)^26^.

For the 10 m walk test, performed to evaluate patients’ locomotor ability, subjects were asked to walk 10 m, and the time taken was measured in seconds. For the 9-hole peg test, the non-dominant hand was tested as a measure of fine motor ability. Patients were asked to take nine pegs from a bowl, insert them into nine holes in a board as quickly as possible, and then remove them one at a time and return them to the container. The time taken for this process was clocked in seconds.

### Electrophysiological examinations

Motor and sensory conduction velocities of the peroneal, tibial, and sural nerves were determined using surface stimulation and recording electrodes. The motor nerve conduction velocities of the peroneal and tibial nerves were determined by stimulation at the knee and ankle while recording the compound muscle action potentials (CMAPs) over the extensor digitorum brevis and adductor hallucis, respectively. CMAPs were measured from baseline to negative peak values. The sensory nerve conduction velocities (NCVs) and sensory nerve action potentials (SNAPs) for the sural nerves were obtained by orthodromic scoring. SNAPs were measured from positive peaks to negative peaks.

### Statistical analysis

Interobserver agreement for the FF and CSA measurements was calculated using the intraclass correlation coefficient (ICC) and interpreted as follows: less than 0.4 indicates poor agreement, 0.40–0.59 indicates moderate agreement, 0.60–0.74 indicates good agreement, and 0.75–1.0 indicates excellent agreement^27^. Comparisons of FF and CSA between the two groups (patients and healthy controls) at each level were conducted using a linear mixed model with adjustment for sides (left or right) and reviewers. The normality of continuous variables was checked using the Shapiro–Wilk test, and FF and CSA were square root transformed due to non-normality before the analysis of the linear mixed model. The FF and CSA at different levels were compared within each group using generalized estimating equations with adjustment for sides and reviewers, and those *p*-values were corrected using the Bonferroni method due to multiple comparisons among different levels. The relationships between the MRI parameters and clinical data were assessed in the CMT1A group using Pearson or Spearman correlation analyses according to the normality of the data. Correlation analyses were performed for average data values acquired from the left and right sciatic nerves. The relationships between the MRI parameters and onset age were assessed using generalized estimating equations adjusted for levels, sides, and reviewers. A correlation analysis between the CSA and FF and the electrophysiologic parameters was also performed with that method and accounting for side^28^. The body mass index (BMI) of the two groups was compared using the Mann–Whitney U test to check for the presence of a significant difference that could affect the FF and CSA of the sciatic nerve. Descriptive statistics for MRI parameters, clinical variables, and demographic variables are presented as the mean ± standard deviation (SD), median (interquartile range [IQR]), and range. Statistical analyses were performed using SAS version 9.4 (SAS Institute, Cary, NC, USA). A two-tailed *p* value < 0.05 was considered statistically significant.

## Results

### Patient characteristics

Each group contained 8 male and 10 female participants aged 20 to 37 years [CMT1A patients, mean ± SD, 30.1 ± 4.3 (range 23–37); volunteers, mean ± SD, 28.2 ± 1.2 (range 20–36)]. Clinical and demographic data for the CMT1A patients are shown in Table 1. The onset age ranged from 5–30 years (median [IQR]; 13.5 [8–15.75]). The CMTNSv2 results ranged from 4 to 21 (median [IQR]; 13.5 [7.25–18.75]). The MRI analysis results are shown in Supplementary Table S2. No significant difference in BMI was found between the two groups (*p* = 0.270; volunteers: median [IQR]; 21.60 [20.60–25.10]; CMT1A patients: median [IQR]; 23.06 [21.48–27.63]).

**Table 1:** Clinical and demographic data for Charcot-Marie-Tooth disease type 1A patients.

Patient number	Sex	Age at exam	Age at onset	CMTNSv2 (0–36)	10 m walk test (s)	9—hole peg test (s)	BMI
1	Male	22	10	4	6.0	20.3	21.0
2	Male	24	22	7	NP	NP	23.9
3	Male	26	26	19	NP	21.7	31.7
4	Male	26	5	18	6.8	28.4	27.7
5	Male	27	8	17	16.0	24.9	25.6
6	Male	29	11	14	15.4	24.1	23.9
7	Male	34	10	11	17.6	30.1	31.7
8	Male	36	7	21	NP	NP	21.1
9	Female	20	15	21	8.1	35.3	22.2
10	Female	22	16	13	7.0	19.5	35.3
11	Female	23	21	7	NP	NP	22.3
12	Female	28	13	20	19.3	24.3	19.1
13	Female	32	30	8	10.0	22.5	22.3
14	Female	35	12	19	21.4	44.9	21.2
15	Female	33	10	6	7.0	20.0	23.9
16	Female	25	8	17	15.7	25.3	28.4
17	Female	34	8	6	NP	NP	21.2
18	Female	31	8	13	18.5	21.4	22.1

*CMTNSv2* Charcot-Marie-Tooth neuropathy score version 2, *BMI* body mass index, *NP* not performed.

### Interobserver agreement for FF and CSA measurements

Interobserver agreement for both the CSA and FF measurements was good to excellent, with ICCs for each level as follows: level 1, 0.6520 and 0.7822; level 2, 0.7976 and 0.8332; and level 3, 0.8302 and 0.8253 for CSA and FF, respectively.

### Statistical comparisons of FF and CSA

**Figure 2:**
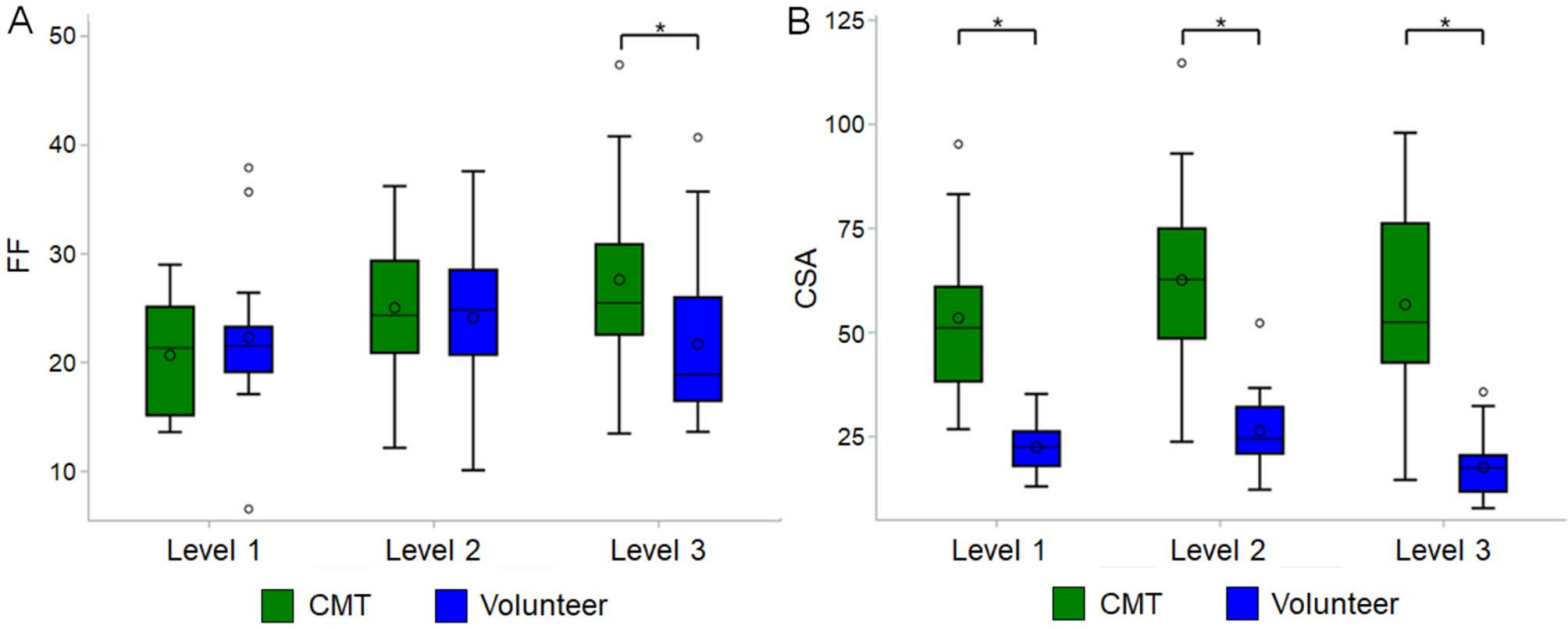
Boxplots comparing the fat fraction and cross-sectional areas of the sciatic nerve between healthy volunteers and Charcot-Marie-Tooth disease type IA patients. An asterisk indicates a significant difference between the two groups. *FF* fat fraction of the sciatic nerve, *CSA* cross-sectional area of the sciatic nerve, *CMT* Charcot-Marie-Tooth disease. The circle within the box is the mean, and the circle outside the box is an outlier. The horizontal line in the box indicates the median value.

**Figure 3:**
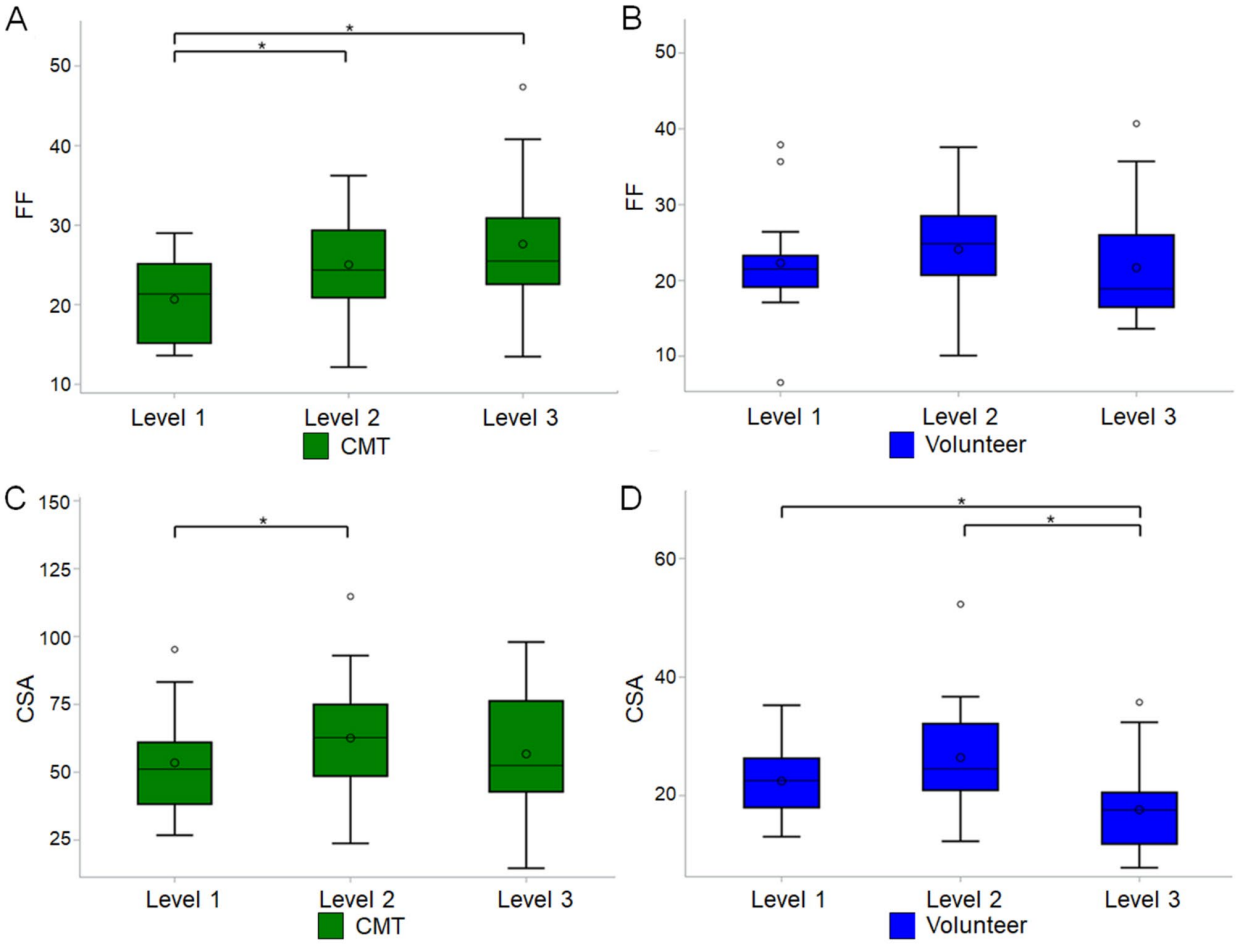
Boxplots comparing the fat fraction and cross-sectional areas at different levels of the sciatic nerve in each group. An asterisk indicates a significant difference between two levels. *FF* fat fraction of the sciatic nerve, *CSA* cross-sectional area of the sciatic nerve, *CMT* Charcot-Marie-Tooth disease. The circle within the box is the mean, and the circle outside the box is an outlier. The horizontal line in the box indicates the median value.

Statistical comparison between groups revealed significantly higher FF values in the patient group at level 3 (Table 2, Fig. 2A,B) and significantly higher CSA in the patient group at all three levels. Comparisons of the FF at different levels in the CMT1A patient group revealed significantly higher values at levels 2 and 3 than at level 1 (Table 3, Fig. 3A). On the other hand, no significant difference by level was seen in the healthy volunteer group (Fig. 3B). Comparisons of the CSA at different levels in the CMT1A patient group showed that level 2 had a significantly higher value than level 1 (Fig. 3C). In the volunteer group, level 3 had a significantly lower value than levels 1 and 2 (Fig. 3D).

**Table 2:** Fat fraction and cross-sectional area of the sciatic nerve compared between Charcot-Marie-Tooth disease type 1A (CMT1A) patients and healthy volunteers.

Parameter	Level	Estimated difference*	Standard error	p value
FF**	1	− 0.1618	0.2259	0.4755
	2	0.1083	0.2582	0.6757
	3	0.6127	0.2630	0.0217^†^
CSA**	1	2.5225	0.3148	< 0.0001^†^
	2	2.7477	0.4000	< 0.0001^†^
	3	3.2614	0.4494	< 0.0001^†^

Analyzed using linear mixed model: group (CMT1A patients, healthy volunteers, sides [L/R]) and reviewers are treated as fixed effects, and subjects are considered as random effects.

*FF* fat fraction of the sciatic nerve, *CSA* cross-sectional area of the sciatic nerve.

*Volunteer group used as reference.

**Square root transformations were performed prior to statistical comparison.

^†^Indicates statistical significance.

**Table 3:** Comparisons of fat fraction and cross-sectional area at different level among Charcot-Marie-Tooth disease type 1A patients and healthy volunteers.

Group	Comparison	Fat fraction			Cross-sectional area		
		Estimate	Standard error	p value	Estimate	Standard error	p value
CMT1A patients	Level 1 vs. 2^†^	4.3676	1.4212	0.0064^§^	9.1118	2.5669	0.0012^§^
	Level 1 vs. 3^†^	6.9519	1.4818	< 0.0001^§^	3.2417	3.7392	1.0000
	Level 2 vs. 3^‡^	2.5843	1.6987	0.3845	− 5.8701	3.2702	0.2179
Volunteers	Level 1 vs. 2^†^	1.8128	1.3758	0.5629	3.9542	2.5211	0.3504
	Level 1 vs. 3^†^	− 0.6057	1.7962	1.0000	− 4.8453	1.6050	0.0076^§^
	Level 2 vs. 3^‡^	− 2.4185	1.8185	0.5506	− 8.7994	2.1882	0.0002^§^

Analyzed using generalized estimating equation with adjustment for sides (L/R) and reviewers.

*Corrected with Bonferroni method due to multiple comparisons.

^†^Level 1 used as reference.

^‡^Level 2 used as reference.

^§^Indicates statistical significance.

### Correlation between CSA and clinical parameters

**Figure 4:**
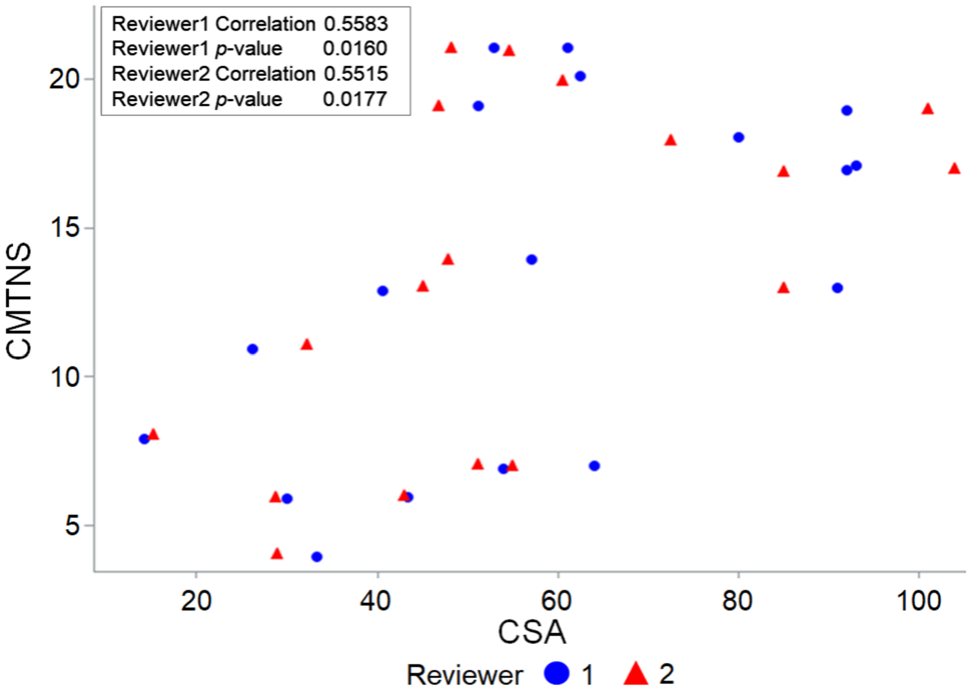
Scatterplots of clinical parameters and cross-sectional areas in Charcot-Marie-Tooth disease patients. CMTNSv2 correlated positively with the CSA measured at level 3 by both reviewers. Blue circles and red triangles represent data analyzed by reviewers 1 and 2, respectively. CMTNSv2, Charcot-Marie-Tooth neuropathy score version 2; CSA, cross-sectional area of the sciatic nerve.

The CSA at level 3 measured by both reviewers correlated positively with CMTNSv2 (r = 0.5583 and 0.5515, *p* = 0.0160 and 0.0177, respectively) (Table 4, Fig. 4). The CSA at level 3 measured by one reviewer correlated significantly with the 10 m walk test time (r = 0.5789, *p* = 0.0382), and that measured by the other reviewer showed a near-significant result (*p* = 0.0638) with a similar trend. The CSA measured by one reviewer at levels 1 and 3 correlated positively with the 9-hole peg test time, but that measured by the other reviewer did not show a significant correlation. A significant negative correlation was present between onset age and CSA (estimate =  − 1.168, *p* = 0.0213) (Table 5).

**Table 4:** Correlation analysis between cross-sectional areas and fat fraction results and clinical parameters in Charcot-Marie-Tooth disease type 1A patients.

Level	Reviewer	Imaging parameter	CMTNSv2*		9–hole peg test time**		10 m walk test time*	
			Correlation coefficient	p value	Correlation coefficient	p value	Correlation coefficient	p value
1	1	CSA	0.2708	0.2770	0.4681	0.0914	0.2420	0.4257
		FF	− 0.2624	0.2928	0.1253	0.6696	0.1316	0.6683
	2	CSA	0.2442	0.3289	0.5473	0.0428^†^	0.2971	0.3243
		FF	− 0.2723	0.2743	0.1560	0.5942	0.1503	0.6241
2	1	CSA	0.4469	0.0630	0.3626	0.2026	0.3920	0.1852
		FF	0.1258	0.6190	0.0330	0.9109	0.3963	0.1801
	2	CSA	0.4652	0.0517	0.4725	0.0880	0.3729	0.2095
		FF	0.0077	0.9757	0.0198	0.9465	0.3848	0.1942
3	1	CSA	0.5583	0.0160^†^	0.4158	0.1392	0.5277	0.0638
		FF	0.0782	0.7577	− 0.0857	0.7708	0.0740	0.8100
	2	CSA	0.5515	0.0177^†^	0.5429	0.0449^†^	0.5789	0.0382^†^
		FF	0.0960	0.7047	0.0242	0.9346	0.0901	0.7698

*CMTNSv2* Charcot-Marie-Tooth neuropathy score version 2, *CSA* cross-sectional area of the sciatic nerve, *FF* fat fraction of the sciatic nerve.

*Analyzed using Pearson correlation analysis.

**Analyzed using Spearman correlation analysis.

^†^Indicates statistical significance.

**Table 5:** Association between fat fraction and cross-sectional area and onset age in Charcot-Marie-Tooth disease type 1A patients.

	Estimate	Standard error	95% confidence limits	p value
FF	0.1632	0.1150	− 0.0621 to 0.3885	0.1558
CSA	− 1.168	0.5072	− 2.1621 to − 0.1739	0.0213^†^

*FF* fat fraction of the sciatic nerve, *CSA* cross-sectional area of the sciatic nerve.

*Analyzed using generalized estimating equation with adjustment for levels, sides (L/R), and reviewers.

^†^Indicates statistical significance.

### Correlation between CSA and electrophysiologic parameters

**Figure 5:**
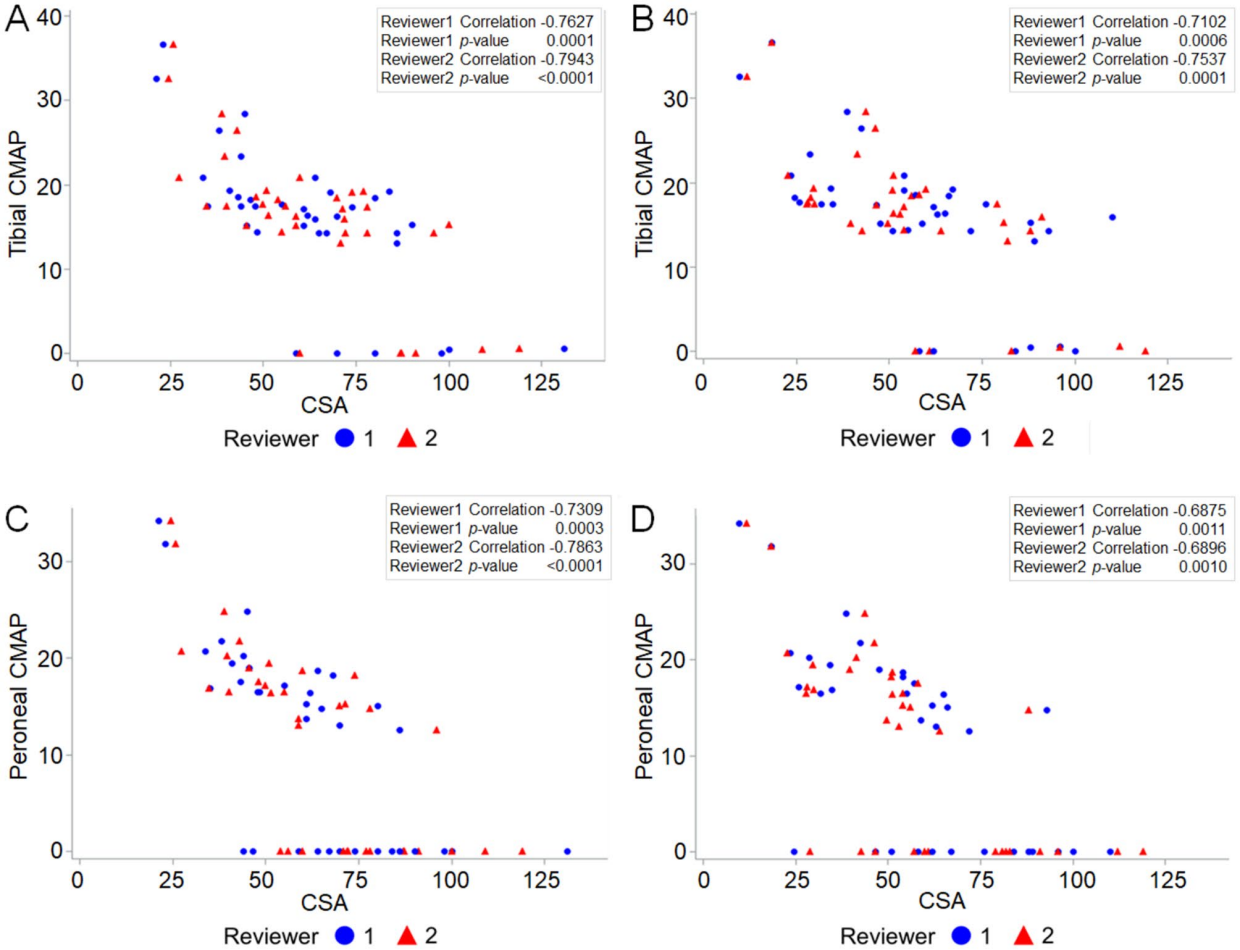
Scatterplots of electrophysiologic parameters and cross-sectional areas in Charcot-Marie-Tooth disease type IA patients. Tibial CMAP ((**A,B**); levels 2 and 3) and peroneal CMAP ((**C,D**); levels 2 and 3) correlated negatively with CSA as measured by both reviewers at levels 2 and 3. Blue circles and red triangles represent data analyzed by reviewers 1 and 2, respectively. *CMAP* compound muscle action potential, *CSA* cross-sectional area of the sciatic nerve.

The electrophysiologic study results are shown in Supplementary Table S3, and its correlation results with the MRI parameters are summarized in Supplementary Table S4 and Fig. 5. Tibial CMAP correlated negatively with CSA as measured by both reviewers at all three levels (*p* < 0.05, r =  − 0.4873 to − 0.7943). Peroneal CMAP correlated negatively with CSA as measured by both reviewers at levels 2 and 3 (*p* < 0.05, r =  − 0.6875 to − 0.7863). Peroneal NCV correlated negatively with CSA at level 3 as measured by one reviewer (*p* = 0.0277, r =  − 0.5142), and that measured by the other reviewer at the same level showed a near significant result (*p* = 0.0519) with a similar trend.

## Discussion

We found significantly higher FF values for the sciatic nerve at level 3, the most distal of the measured levels, in the patient group compared with the control group. Comparisons among FFs measured at different levels within the CMT1A patient group showed that levels 2 and 3 had significantly higher values than level 1. Our results could imply that CMT1A patients have higher than normal intraepineurial fat within the sciatic nerve. It would be beneficial in future studies to conduct a histologic evaluation of intraepineurial fat and nerve myelination in CMT1A to elucidate the potential link between them. Furthermore, Dixon-based fat quantification MRI could be used to evaluate the intraepineurial fat component when studying the effects of a lipid-enriched diet, as has been tried in animal models of CMT1A^11^, which could expand understanding of the disease.

We assumed that increased FF of the sciatic nerve was attributable to interfascicular fat component since interfascicular fat constitutes the major portion of fat tissue within the epineurium. A previous study by Goedee et al.^29^ implied that the portion of nerve fascicle CSA per nerve could be increased in CMT1A patients. One possible explanation for this could be the difference of peripheral nerve that were analyzed. Whereas we evaluated the sciatic nerve which contains a considerable amount of fat^30^, their study focused on smaller peripheral nerves (e.g., median, ulnar, and fibular nerves). The degree of contribution for nerve fascicles and interfascicular compartment regarding increased CSA in various peripheral nerves could be an interesting topic for future research, which may help expand the knowledge on the disease process of CMT1A.

Our result showing a distal tendency for increased FF in CMT1A patients could suggest a distal predominance of increased interfascicular fat. Vaggemose et al.^17^ suggested that interfascicular fat tissue in distal peripheral nerves could be more pronounced in CMT1A patients than in healthy controls based on a quantitative MRI data comparison between the sciatic and tibial nerves. However, their suggestion was derived from proton spin density measured from the nerve, which is not the direct fat quantification we used in our study. Our results from Dixon-based proton density fat quantification, which is a more direct method for measuring FF within a designated area, demonstrate that the distal level of the sciatic nerve had significantly higher FF than the more proximal level in CMT1A patients. On the other hand, no such difference in FF of the sciatic nerve at different levels was identified among the healthy control subjects. Although it would be interesting to examine the FF of more distal nerve segments, including the nerve structure in the lower leg, they were not evaluated in this study because we found no appropriate anatomical landmark distal to that level, and we expected reliable measurement to be limited by their small size. Considering distal predominancy of CMT1A^31^, a future study using dedicated MRI examining unilateral lower limb could provide a better image with higher resolution for evaluating distal nerve segments. Given the absence of histologic correlation in our study, the following could be a premature speculation, but our result could suggest a correlation between increased interfascicular fat and length-dependent distal axonal degeneration in CMT1A patients. Clearly, further study is warranted to verify our findings and find clinical implication of FF, especially considering that FF did not correlate meaningfully with clinical parameters.

In contrast to FF, CSA at level 3, as measured by both reviewers, correlated significantly and positively with CMTNSv2. Previous studies reported a positive correlation between the CSA in several superficial peripheral nerves and CMTNSv2 and suggested the potential value of CSA as an imaging biomarker in CMT1A patients^4,7,9^. The relationship between nerve CSA and disease severity might be explained by the link between the extent of pathologic change, such as onion bulb formation resulting from repeated demyelination–remyelination cycles, and clinical manifestations^7^. The sciatic nerve, although closely related to the clinical manifestation of distal lower limb muscle wasting in CMT1A, has not been deeply investigated in terms of a potential link between nerve CSA and clinical severity or the most relevant level for measurement. Some studies have reported a meaningful correlation between ambulatory function test results and MRI parameters from the thigh muscle^32,33^, but to our knowledge, little has been reported about the potential correlation between sciatic nerve CSA and ambulatory function test results. Our results suggest that sciatic nerve CSA could have potential value as a quantitative imaging biomarker in CMT1A patients and that measurements made at level 3, the gluteus maximus tendon insertion level, could be the most useful. It would be beneficial to confirm the true significance of these imaging parameters in a large cohort of CMT1A patients in the future.

CMAP amplitude, a marker of motor axonal loss, has been reported to correlate with clinical impairment and disability in CMT1A patients^2,34^. Our results indicate that the CSA of the sciatic nerve correlates negatively with tibial and peroneal CMAP amplitude. Previous studies using ultrasound to evaluate the median nerves in CMT1A patients found significant negative correlations between NCV and CSA, whereas negative correlations between CMAP or SNAP amplitude and CSA were inconsistently demonstrated^7,35^. We assume that our result is in agreement with a previous study finding that increased peripheral nerve CSA in CMT1A patients reflects axonal loss and the progress of demyelination.

We found significantly higher sciatic nerve CSA in the patient group than the control group at all three measured levels. Whereas the CSA comparison between levels in the normal subjects showed significantly smaller CSAs at the distal level, CMT1A patients showed significantly larger CSA at level 2 than at level 1. In other words, the normal anatomical tapering of CSA in the sciatic nerve is absent in CMT1A patients, and in fact, the reverse is true. Few peripheral nerve CSA measurements at multiple levels have been compared between normal subjects and CMT1A patients, nor have previous comparisons considered differences between levels among CMT1A patients. A CSA analysis of certain peripheral nerve structures at multiple levels in CMT1A patients that also analyzes the relationships among the values from different levels could be an interesting subject for future studies.

A significant negative correlation was demonstrated between onset age and sciatic nerve CSA. In our results, a 1-year decrease in the age of onset correlated with a 1.168 mm^2^ increase in the sciatic nerve CSA. Little has been reported on the potential relationship between the onset age of CMT1A patients and nerve CSA. It is known that first symptoms generally appear during childhood or adolescence in CMT1A, but patients present with widely varying disease severity^1^. It is unclear whether the progression rate of the disease is constant^1,36^. It would be interesting in future studies to investigate longitudinal changes in sciatic nerve CSA and their potential link with clinical progression of the disease.

A preliminary study by Ratner et al.^25^ measured the CSA and FF of the sciatic nerve in normal subjects at the level of the ischial tuberosity and lesser trochanter, which are similar to levels 1 and 2 in our study, respectively. They reported that their measurements of both CSA and FF were reproducible in terms of interobserver agreement, which is also in agreement with our results. Our study also showed excellent inter-rater agreement for measurements at level 3, which is more distal than levels 1 and 2.

Previous studies have suggested that age and sex have a significant relationship with nerve FF and CSA^15,25^. Our study used data from age- and sex-matched case–control subjects, which means that our results are free from that concern. Although not clearly demonstrated by previous study, a potential relationship between obesity and the FF and CSA of peripheral nerves has been suggested^25,37^. Our statistical comparison of BMI between the patient and volunteer groups revealed no significant difference in our study.

Our study has several limitations. First, it was conducted with a small number of patients. Second, we did not perform a histologic analysis through nerve biopsies, so we could not evaluate the pathologic significance of the increased FF within the sciatic nerve. However, our study provides imaging evidence that the peripheral nerves of CMT1A patients could show increased intraepineurial fat, warranting further study on its possible effect in pathogenesis. Third, distal nerve segments were not evaluated as described above. Fourth, measurement errors could have occurred in our analyses because peripheral nerves are smaller than other anatomic structures. Fifth, our subjects were in their 20 s and 30 s, and the CMTNSv2 of the patient group ranged from 4 to 21, which implies that our patients were in mild to moderate stages of the disease. It would be interesting to perform longitudinal analyses in patients with a wider distribution of ages and disease stages. Sixth, FF did not show meaningful correlation with clinical parameters. We believefurther study is warranted to investigate FF of more distal nerve segment for their possible correlation with clinical parameters in larger study population.

In conclusion, we found a significantly higher FF at level 3, the most distal of the measured levels, in the sciatic nerves of CMT1A patients compared with those of the controls. Comparisons of the FF measured at different levels within the CMT1A patient group showed significantly higher values for levels 2 and 3 than for level 1, whereas no such difference was seen in the controls. Although this could suggest the presence of increased intraepineurial fat in the sciatic nerves of CMT1A patients, with a possible distal tendency, future investigation would be mandatory to find clinical significance and implication of increased FF. The CSA measured at level 3 by both reviewers correlated significantly and positively with CMTNSv2. These results may imply that the sciatic nerve CSA at level 3 has potential value as an imaging marker for clinical severity in CMT1A patients. Further studies are required to confirm our findings.

## Supplementary Information

Supplementary Information.

## Abbreviations

CMT: Charcot-Marie-Tooth disease
PMP: Peripheral myelin protein
CSA: Cross-sectional area
MRI: Magnetic resonance imaging
FF: Fat fraction
ROI: Region of interest
CMTNSv2: CMT neuropathy score version 2
CMAP: Compound muscle action potential
NCV: Nerve conduction velocity
SNAP: Sensory nerve action potential
IQR: Interquartile range
SD: Standard deviation
ICC: Intraclass correlation coefficient
BMI: Body mass index
