# Supplementary material for: Intraepineurial fat quantification and cross-sectional area analysis of the sciatic nerve using MRI in Charcot-Marie-Tooth disease type 1A patients
Source: Sci Rep. 2021 Nov 2;11:21535. doi: 10.1038/s41598-021-00819-0 (PMC8563983; doi:10.1038/s41598-021-00819-0)
Supplement: Supplementary file 1 — Supplementary Information. [file 41598_2021_819_MOESM1_ESM.docx]

**Supplementary Table S1.** Summary of MRI parameters

| Parameters | mDixon-Quant | T1-Weighted TSE Imaging in Axial Plane | T1-Weighted TSE Imaging in Coronal Plane | T2-Weighted Dixon Imaging in Axial Plane |
| --- | --- | --- | --- | --- |
| Repetition time (ms) | 6.6 | 613.7 | 15 | 4635.5 |
| Echo time (ms) | 1.01, 1.91, 2.81, 3.71, 4.61, 5.51 | 16.7 | 5 | 80 |
| Number of signals averaged | 2 | 1 | 1 | 1 |
| Matrix size | 192 x 192 | 320 x 320 | 320 x 317 | 320 x 320 |
| Field of view (mm) | 350 x 350 | 350 x 350 | 350 x 350 | 350 x 350 |
| Slice thickness (mm) | 6 | 2 | 5 | 2 |
| Gap (mm) | 0 | 1 | 0.5 | 1 |
| Reconstructed voxel size (mm) | 1.82 | 0.68 | 0.68 | 0.68 |
| Number of slices | 140 | 67 | 25 | 67 |
| Imaging time (sec) | 72.5 | 162 | 200 | 194 |

mDixon-Quant, 3D multiple gradient echo Dixon-based sequence for fat quantification; TSE, turbo-spin echo

**Supplementary Table S2.** Fat fraction and cross-sectional areas of the sciatic nerves of Charcot-Marie-Tooth disease type IA patients and healthy volunteers

| Group | Parameter | Level | Reviewer 1 | | Reviewer 2 | |
| --- | --- | --- | --- | --- | --- | --- |
|  |  |  | Left* | Right* | Left* | Right* |
| CMT patients | FF (%) | 1 | 22.50±6.38  (24.50, 16.60–26.84) | 18.44±5.29  (16.80, 14.86–22.21) | 22.90±6.30  (23.32, 17.42–27.94) | 18.84±5.84  (18.21, 14.06–22.73) |
|  |  | 2 | 26.00±7.82  (24.86¸21.83–30.73) | 24.05±6.97  (24.90, 20.18–29.85) | 25.52±8.44  (22.96, 20.21–29.87) | 24.58±7.12  (24.10¸19.46–30.95) |
|  |  | 3 | 29.23±10.04  (27.30, 22.05–34.26) | 25.84±8.09  (25.55, 19.72–29.90) | 29.24±9.29  (27.02, 24.17–33.11) | 26.18±8.90  (23.91, 20.89–30.33) |
|  | CSA (mm^2^) | 1 | 53.57±22.59  (48.50, 38.05–65.50) | 54.15±18.12  (51.50, 42.35–56.50) | 53.57±19.14  (55.00, 36.85–63.75) | 52.74±21.05  (47.65, 40.40–57.64) |
|  |  | 2 | 63.92±25.27  (62.50, 47.95–73.00) | 60.08±21.51  (61.50, 44.03–77.50) | 64.45±25.16  (60.00, 48.58–78.00) | 62.03±21.85  (57.50, 46.88–73.53) |
|  |  | 3 | 59.68±28.49  (57.50, 40.63–81.75) | 55.66±21.97  (56.50¸36.73–70.75) | 58.92±29.47  (54.00, 44.50–76.00) | 52.74±21.37  (50.35, 40.25–63.00) |
| Volunteers | FF (%) | 1 | 22.21±6.50  (21.79, 18.32–26.10) | 21.54±7.53  (20.67, 18.00–23.15) | 22.91±6.74  (22.48, 18.96–26.04) | 22.52±8.43  (21.47, 19.38–23.63) |
|  |  | 2 | 24.67±7.89  (25.16, 20.11–30.25) | 23.49±8.58  (23.05, 17.43–28.01) | 24.59±8.16  (24.64, 20.24–30.00) | 23.69±8.46  (24.25¸18.29–27.46) |
|  |  | 3 | 24.08±8.33  (22.72, 17.96–28.13) | 19.03±7.88  (15.85, 14.03–21.54) | 24.56±8.91  (24.52, 17.06–28.90) | 19.09±7.84  (16.37¸ 14.00–23.26) |
|  | CSA (mm^2^) | 1 | 22.82±7.98  (21.85, 19.15–27.85) | 21.89±7.72  (21.20, 17.93–23.65) | 22.89±6.92  (22.51, 19.45–26.83) | 22.23±7.60  (21.35¸17.48–24.03) |
|  |  | 2 | 25.07±10.22  (23.15, 18.98–31.00) | 26.73±11.72  (24.70, 17.38–31.70) | 25.87±10.91  (24.65, 18.45–32.13) | 27.99±12.33  (24.05, 20.08–33.80) |
|  |  | 3 | 17.94±8.83  (16.15, 10.45–23.88) | 16.73±8.32  (14.75, 11.13–20.53) | 18.60±9.84  (16.15, 11.53–23.48) | 17.19±7.95  (16.30, 11.25–22.51) |

^*^ Data are median ± standard deviation, with interquartile ranges in parentheses.

FF, fat fraction of the sciatic nerve; CSA, cross-sectional area of the sciatic nerve.

**Supplementary Table S3.** Electrophysiologic study results for Charcot-Marie-Tooth disease patients

| Patient number | Peroneal NCV (m/s)  (left/right) | Peroneal CMAP (mV)  (left/right) | Tibial NCV (m/s)  (left/right) | Tibial CMAP (mV)  (left/right) | Sural NCV (m/s)  (left/right) | Sural SNAP (mV)  (left/right) |
| --- | --- | --- | --- | --- | --- | --- |
| 1 | 0.9/2.6 | 16.5/16.9 | 3.2/4.7 | 17.4/17.4 | A/A | A/A |
| 2 | 0.4/1.2 | 18.7/18.2 | 7.8/8.0 | 20.8/19.1 | A/A | A/A |
| 3 | 1.5/0.6 | 16.5/19.0 | 0.5/0.3 | 14.4/15.2 | A/A | A/A |
| 4 | A/1.4 | A/12.6 | 2.4/1.2 | 15.3/14.2 | A/A | A/A |
| 5 | A/A | A/A | 0.5/0.2 | 15.9/17.4 | A/A | A/A |
| 6 | 1.6/A | 13.0/A | 0.2/0.6 | 16.2/14.3 | A/A | A/A |
| 7 | 2.7/2.7 | 20.8/20.2 | 0.7/3.3 | 20.8/23.4 | 2.1/1.1 | 19.4/19.7 |
| 8 | 0.8/0.6 | 17.6/16.4 | 2.3/2.0 | 18.6/16.4 | A/A | A/A |
| 9 | A/0.3 | A/13.7 | 0.3/0.9 | 17.3/15.1 | A/A | A/A |
| 10 | 1.1/1.8 | 24.9/21.8 | 4.9/6.3 | 28.4/26.5 | A/A | A/A |
| 11 | 0.8/0.8 | 15.3/15.1 | 3.6/4.1 | 17.1/18.5 | A/A | A/A |
| 12 | A/A | A/A | 0.2/0.4 | A/19.2 | A/A | A/A |
| 13 | 4.2/3.1 | 34.2/31.8 | 10.0/3.3 | 32.6/36.6 | 3.0/4.4 | 25.9/27.5 |
| 14 | A/A | A/A | A/A | A/A | A/A | A/A |
| 15 | 22.9/4.6 | 17.2/19.5 | 7.7/7.7 | 17.6/19.3 | A/A | A/A |
| 16 | A/A | A/A | 13.7/10.0 | 0.6/0.5 | A/A | A/A |
| 17 | A/A | A/A | A/1.0 | A/18.2 | A/A | A/A |
| 18 | 0.2/A | 14.8/A | 0.7/1.8 | 14.3/13.1 | A/A | A/A |

NCV, nerve conduction velocity; CMAP, compound muscle action potential; SNAP, sensory nerve action potential; A, absent

**Supplementary Table S4.** Correlations between cross-sectional areas and fat fraction results and electrophysiologic study parameters in Charcot-Marie-Tooth disease type IA patients

| Electrophysiologic study parameter | MRI parameter | Level | Correlation coefficient  (R1/R2) | 95% Confidence limits  (R1/R2) | *p* value  (R1/R2) |
| --- | --- | --- | --- | --- | --- |
| Peroneal NCV | CSA | 1 | -0.4182 / -0.3995 | (-0.7405, 0.0605) / (-0.7302, 0.0829) | 0.0845 / 0.1014 |
|  |  | 2 | -0.2790 / -0.3630 | (-0.6599, 0.2160) / (-0.7096, 0.1251) | 0.2670 / 0.1407 |
|  |  | 3 | -0.5142 / -0.4636 | (-0.7911, -0.0622) / (-0.7649, 0.0042) | 0.0277^†^ / 0.0519 |
|  | FF | 1 | 0.2931 / 0.1388 | (-0.2014, 0.6685) / (-0.3508, 0.5688) | 0.2423 / 0.5885 |
|  |  | 2 | -0.0493 / -0.0585 | (-0.5045, 0.4274) / (-0.5114, 0.4199) | 0.8486 / 0.8207 |
|  |  | 3 | 0.1020 / 0.0564 | (-0.3832, 0.5430) / (-0.4216, 0.5098) | 0.6919 / 0.8270 |
| Peroneal CMAP | CSA | 1 | -0.3150 / -0.2804 | (-0.6817, 0.1780) / (-0.6608, 0.2145) | 0.2066 / 0.2644 |
|  |  | 2 | -0.7309 / -0.7863 | (-0.8930, -0.4007) / (-0.9167, -0.5046) | 0.0003^†^ / <0.0001^†^ |
|  |  | 3 | -0.6875 / -0.6896 | (-0.8739, -0.3249) / (-0.8748, -0.3286) | 0.0011^†^ / 0.0010^†^ |
|  | FF | 1 | 0.3052 / 0.3346 | (-0.1886, 0.6758) / (-0.1567, 0.6932) | 0.2221 / 0.1777 |
|  |  | 2 | 0.2494 / 0.1994 | (-0.2461, 0.6416) / (-0.2949, 0.6095) | 0.3237 / 0.4337 |
|  |  | 3 | 0.2620 / 0.1787 | (-0.2335, 0.6494) / (-0.3144, 0.5959) | 0.2989 / 0.4842 |
| Tibial NCV | CSA | 1 | -0.0188 / -0.1606 | (-0.4814, 0.4520) / (-0.5837, 0.3311) | 0.9420 / 0.5305 |
|  |  | 2 | 0.2252 / 0.1273 | (-0.2701, 0.6262) / (-0.3610, 0.5609) | 0.3749 / 0.6200 |
|  |  | 3 | -0.1517 / 0.0313 | (-0.5777, 0.3392) / (-0.4420, 0.4910) | 0.5538 / 0.9034 |
|  | FF | 1 | -0.0224 / 0.0234 | (-0.4842, 0.4492) / (-0.4484, 0.4850) | 0.9310 / 0.9278 |
|  |  | 2 | -0.1172 / -0.2864 | (-0.5538, 0.3699) / (-0.6644, 0.2084) | 0.6484 / 0.2539 |
|  |  | 3 | -0.1529 / -0.1326 | (-0.5785, 0.3381) / (-0.5645, 0.3564) | 0.5506 / 0.6055 |
| Tibial CMAP | CSA | 1 | -0.4982 / -0.5487 | (-0.7829, -0.0408) / (-0.8085, -0.1101) | 0.0342^†^ / 0.0169^†^ |
|  |  | 2 | -0.7627 / -0.7943 | (-0.9067, -0.4594) / (-0.9200, -0.5204) | 0.0001^†^ / <0.0001^†^ |
|  |  | 3 | -0.7102 / -0.7537 | (-0.8840, -0.3640) / (-0.9029, -0.4425) | 0.0006^†^ / 0.0001^†^ |
|  | FF | 1 | -0.2143 / -0.1124 | (-0.6192, 0.2807) / (-0.5504, 0.3741) | 0.3993 / 0.6620 |
|  |  | 2 | -0.1451 / -0.0984 | (-0.5732, 0.3451) / (-0.5404, 0.3863) | 0.5713 / 0.7024 |
|  |  | 3 | 0.1009 / 0.1337 | (-0.3841, 0.5422) / (-0.3554, 0.5653) | 0.6950 / 0.6025 |
| Sural NCV | CSA | 1 | 0.2384 / 0.1220 | (-0.2571, 0.6346) / (-0.3657, 0.5571) | 0.3466 / 0.6349 |
|  |  | 2 | -0.3824 / -0.4878 | (-0.7206, 0.1028) / (-0.7776, -0.0271) | 0.1187 / 0.0390^†^ |
|  |  | 3 | -0.3289 / -0.3819 | (-0.6899, 0.1630) / (-0.7204, 0.1034) | 0.1858 / 0.1192 |
|  | FF | 1 | 0.2249 / 0.1899 | (-0.2704, 0.6260) / (-0.3039, 0.6033) | 0.3755 / 0.4565 |
|  |  | 2 | 0.1276 / 0.3686 | (-0.3607, 0.5611) / (-0.1187, 0.7128) | 0.6192 / 0.1341 |
|  |  | 3 | 0.2399 / 0.3071 | (-0.2556, 0.6356) / (-0.1865, 0.6769) | 0.3433 / 0.2190 |
| Sural SNAP | CSA | 1 | 0.4261 / 0.2822 | (-0.0510, 0.7448) / (0.2127, 0.6619) | 0.0780 / 0.2612 |
|  |  | 2 | 0.0194 / 0.0217 | (-0.4515, 0.4819) / (-0.4497, 0.4837) | 0.9400 / 0.9330 |
|  |  | 3 | 0.1081 / 0.1260 | (-0.3779, 0.5473) / (-0.3622, 0.5599) | 0.6743 / 0.6237 |
|  | FF | 1 | -0.0763 / -0.0761 | (-0.5245, 0.4050) / (-0.5243, 0.4052) | 0.7673 / 0.7678 |
|  |  | 2 | 0.0089 / 0.2430 | (-0.4599, 0.4738) / (-0.2525, 0.6375) | 0.9725 / 0.3369 |
|  |  | 3 | 0.0183 / 0.1224 | (-0.4525, 0.4810) / (-0.3654, 0.5574) | 0.9436 / 0.6338 |

^*^ Analyzed with adjustment for sides (left/right).

^†^ Indicates statistical significance.

NCV, nerve conduction velocity; CMAP, compound muscle action potential; SNAP, sensory nerve action potential; R1 and R2, reviewer 1 and reviewer 2; CSA, cross-sectional area of the sciatic nerve; FF, fat fraction of the sciatic nerve
